# Anti‐wrinkle efficacy of standardized phenolic acids polymer extract (PAPE) from propolis: Implications for antiaging and skin health

**DOI:** 10.1111/jocd.16405

**Published:** 2024-06-28

**Authors:** Božo Radić, Saša Radić, Tomislav Mašek, Jelena Šuran

**Affiliations:** ^1^ Apiotix Technologies d.o.o. Split Croatia; ^2^ Department of Animal Nutrition and Dietetics, Faculty of Veterinary Medicine University of Zagreb Zagreb Croatia

**Keywords:** antiaging products, clinical testing, retinol, skin‐care products

## Abstract

**Background:**

The increasing quest for effective and safe antiaging skincare solutions has led to a surge in the exploration of natural compounds such as phenolic acids. Despite the proven efficacy of traditional antiaging ingredients like retinol, their associated side effects have necessitated the search for alternatives.

**Aims:**

This study aimed to assess the anti‐wrinkle efficacy of a standardized phenolic acids polymer extract (PAPE) from propolis, employing both in vitro and clinical methodologies to explore its suitability as a novel antiaging skincare ingredient for sensitive and nonsensitive skin types.

**Patients/Methods:**

The study comprised of evaluating PAPE effects on key skin health biomarkers in dermal fibroblasts and keratinocytes. A double‐blind, randomized clinical trial involving female participants aged 30–70 years assessed the wrinkle‐reducing effectiveness of face creams formulated with two concentrations of PAPE (1.5% and 3%) over a 28‐day period.

**Results:**

In vitro studies indicated that PAPE could modulate inflammation and tissue remodeling biomarkers. The clinical trial demonstrated that applying PAPE‐enriched cream resulted in significant wrinkle reduction, with 25% and 34% improvements for the 1.5% and 3% PAPE formulations, respectively. Subjective feedback from participants further validated the antiaging efficacy and overall satisfaction with the product.

**Conclusion:**

Incorporating PAPE offers a compelling antiaging solution, significantly reducing wrinkle depth with a favorable safety profile. The study substantiates PAPE's potential as an effective and safe alternative to conventional antiaging ingredients, aligning with the cosmetic industry's shift toward natural, evidence‐based formulations.

## 1 INTRODUCTION

### 1.1 Background

The pursuit of effective antiaging skincare products is driven by the need to address the complex skin aging process, influenced by intrinsic and extrinsic factors. Retinol has traditionally been a key component in antiaging formulations because it inhibits UV‐induced matrix metalloproteinases (MMPs) and promotes collagen synthesis in photoaged skin.^1^ Since retinol has several side effects, such as dryness, rash, and soreness,^2^ the search for alternative compounds and innovative formulations continues. Natural compounds such as bakuchiol have emerged as promising candidates, offering retinol‐like functional properties without the typical irritant side effects.^3^ Additionally, hyaluronic acid has demonstrated efficacy in maintaining skin hydration and reducing the appearance of wrinkles. Peptides, such as acetylhexapeptide‐3 (AHP‐3) and palmitoyl pentapeptide‐4 (PPP‐4), have also shown promise in stimulating collagen and elastin production, making them potentially effective topical antiaging agents.^4^ As the skincare industry evolves, developing antiaging products becomes increasingly crucial, with a growing emphasis on green and sustainable formulations to meet consumer demand.^5^ Experimenting with novel ingredients, advanced delivery systems, and evidence‐based efficacy assessments contributes to advancing antiaging skincare, catering to consumers' evolving needs and preferences.

The pursuit of effective anti‐wrinkle solutions has also sparked interest in the potential of phenolic acids in skincare. Phenolic acids, such as ferulic acid, caffeic acid, and others, have been identified as essential cosmeceutical components with promising anti‐wrinkling properties.^6^ These compounds have been found to be protective in maintaining the integrity of key skin structures, including keratinocytes, fibroblasts, collagen, and elastin.^7^ Phenolic acids have demonstrated antioxidant properties, which are crucial for combating oxidative stress and preventing skin aging, and the inhibition of MMPs, enzymes involved in skin aging processes.^7^ The efficacy of phenolic acids in skincare formulations has been attributed to their ability to penetrate the skin epidermis and dermis, indicating their potential for topical application.^8^ Additionally, they have been associated with anti‐inflammatory effects, further contributing to their potential in addressing skin aging concerns.^9^

Propolis, a natural resinous substance collected by honeybees from tree buds, is a rich source of phenolic acids such as caffeic acid, ferulic acid, and p‐coumaric acid, which are essential for its antioxidant, anti‐inflammatory, and antimicrobial properties, making it a valuable candidate for skin health applications^10^, ^11^ Propolis potential in promoting wound healing, tissue regeneration, and collagen synthesis further underscores its suitability as a source of bioactive ingredients for skincare formulations.^12^, ^13^, ^14^, ^15^

The greatest challenge of propolis utilization in skincare formulations at optimal concentrations for desired effects is variability in composition, which stems from differences in botanical and geographical origins.^16^, ^17^ Standardization efforts are crucial for establishing quality control measures and ensuring the reliability of propolis‐derived phenolic acids in skincare formulations. Thus, we developed a method of propolis extraction with polyethylene glycol and standardization using the content of three phenolic acids and caffeic acid phenethyl ester (CAPE).^18^ The result of these two processes is a standardized phenolic acid polymer extract from propolis (PAPE), which can be applied in pharmaceutical, cosmetic, and food formulations.

### 1.2 Objectives

The aim of our study was to test PAPE in vitro, in dermal fibroblasts and keratinocytes models of inflammation, and clinical trial in face cream for its antiaging efficacy in women with sensitive and nonsensitive skin to explore its potential in antiage application.

## 2 METHODS

### 2.1 PAPE characterization and formulations preparation

The initial extract, exhibiting the highest concentration, was produced according to the methodology outlined in Patent No. WO/2020/169425.^18^ This method resulted in an extract comprising 35.3% propolis in PEG 400 solvent. Subsequently, it underwent standardization through dilution and centrifugation in PEG 400, rendering it suitable for in vitro studies and clinical trials. The standardized product from this method was designated as PAPE.

The concentrations of biologically significant markers in both the initial extract and PAPE were quantified using HPLC‐UV, as specified in Patent No. WO/2020/169425.^18^, ^19^ Selected based on prior literature,^20^ the bioactive markers of propolis included caffeic acid (≥98% HPLC), p‐coumaric acid (≥98% HPLC), trans‐ferulic acid (99% HPLC), trans‐cinnamic acid (≥96.5% GC), chrysin (≥98% HPLC), quercetin (≥95% HPLC), apigenin (≥99% HPLC), and kaempferol (≥90% HPLC). These chemically pure standards were used to determine the quantitative presence of these compounds in the propolis extract (sourced from Sigma‐Aldrich).

The aforementioned samples chosen for standardization were prepared for analysis by dilution with ethanol: water mixture, 75:25, V/V, up to the concentration of 100 μg/mL. The samples of liquid propolis extracts (100 μL) were diluted before analysis with ethanol: water mixture, 75:25, V/V (900 μL), in a ratio of 1:10 w/w (10× dilution). Analyses were carried out on the Shimadzu LC201CHT instrument, equipped with an autosampler, pump, degasser, column oven, and UV–VIS detector, under the following conditions: (i) chromatographic column: Ascentis express; C18; dimensions: 15 cm × 3.0 mm; diameter of particles in the column: 2.7 μm; (ii) mobile phase: A = 0.1% formic acid aqueous solution, B = methanol; gradient: 0 min, 80% A, 20% B; 3 min, 70% A, 30% B; 60 min, 20% A, 80% B; 90 min, 20% A, 80% B; 100 min, 70% A, 30% B; 105 min, 80% A, 20% B; (iii) column temperature: 30°C; (iv) flow: 0.25 mL/min; (v) analysis time: 110 min; (vi) wavelength on UV–VIS detector: for detection: 370 nm, for integration: 290 nm; (vii) injection volume: 10 μL; (viii) pressure: 210–290 bars.

For the in vitro study, PAPE was further diluted with phosphate‐buffered saline (PBS) in a 1:8 ratio and centrifuged in between steps to get a clear solution without precipitates for cytokine readouts. For clinical trials, PAPE was mixed with the common base cream (Belobaza®, Belupo, Croatia) to obtain 100 g of skin cream (leave‐on emulsion) with 1.5% (Product A) and 3% (Product B) PAPE.

### 2.2 In vitro study—BioMAP profiling

In vitro studies were performed as a part of BioMAP phenotyping profiling (Eurofins, USA). The panel consists of human primary cell‐based systems designed to model different aspects of the human body in an in vitro format.^21^, ^22^, ^23^, ^24^, ^25^, ^26^, ^27^ Human primary cells in BioMAP systems are used at early passage (Passage 4 or earlier) to minimize adaptation to cell culture conditions and preserve physiological signaling responses. All cells are from a pool of multiple donors (*n* = 2–6), commercially purchased and handled according to the manufacturers' recommendations.

BioMAP systems are constructed with one or more primary cell types from healthy human donors, with stimuli (such as cytokines or growth factors) added to capture relevant signaling networks that naturally occur in human tissue or pathological conditions. Skin biology is addressed in the KF3CT system modeling (human keratinocytes and dermal fibroblasts) Th1 cutaneous inflammation and the HDF3CGF system modeling (human dermal fibroblasts) wound healing.^24^, ^25^

HDF3CGF system models wound healing and matrix/tissue remodeling in the context of Th1‐1 type inflammation. The HDF3CGF system is relevant for various diseases, including fibrosis, rheumatoid arthritis, and psoriasis, as well as stromal biology in tumors. This system was stimulated with interferon‐gamma (IFN‐γ), tumor necrosis factor‐alpha (TNF‐α), IL‐1β, epidermal growth factor (EGF), basic fibroblast growth factor (FGF), and platelet‐derived growth factor‐BB (PDGF‐BB) to model wound healing processes. The measured readouts to evaluate skin repair and regeneration included CCL2/MCP‐1, CD106/VCAM‐1, CD54/ICAM‐1, Collagen I, Collagen III, CXCL10/IP‐10, CXCL11/I‐TAC, CXCL8/IL‐8, CXCL9/MIG, EGFR, M‐CSF, MMP‐1, PAI‐I, SRB, TIMP1, and TIMP‐2.

KF3CT system models Th1‐type cutaneous inflammation and is an environment that promotes monocyte and T cell adhesion and recruitment. The KF3CT system is relevant for cutaneous responses to tissue damage caused by mechanical, chemical, or infectious as well as certain states of psoriasis and dermatitis. This system, using keratinocytes, was stimulated with IL‐1β, IFN‐γ, TGF‐β, and TNF‐α to model Th1‐mediated cutaneous inflammation. The modulation of the inflammatory response by test agents was assessed through the following readouts: CCL2/MCP‐1, CD54/ICAM‐1, CXCL10/IP‐10, CXCL8/IL‐8, CXCL9/MIG, IL‐1α, MMP‐9, TIMP‐2, PAI‐I SRB, TMP‐2, and uPA.

Systems are derived from single‐cell or co‐culture systems and cultured in 96 or 384‐well plates until confluence. The PAPE was prepared in PBS and added at a starting concentration of 164.8 μg/g of sum phenolic acid (ferulic, caffeic acid, CAPE, and *para*‐coumaric) 1‐h before stimulation and remained in culture for 24‐h or as otherwise indicated (other systems, not showed here). Each plate contained drug controls (e.g., legacy control test agent colchicine at 1.1 μM), negative controls (e.g., non‐stimulated conditions), and vehicle controls (e.g., 0.1% DMSO) appropriate for each system. Biomarker levels of cell‐associated and cell membrane targets were measured by direct ELISA. Soluble factors from supernatants were quantified using either HTRF® detection, bead‐based multiplex immunoassay, or capture ELISA. Overt adverse effects of test agents on cell proliferation and viability (cytotoxicity) were detected by sulforhodamine B (SRB) staining for adherent cells and alamarBlue® reduction for cells in suspension.

#### 2.2.1 Data analysis

Biomarker measurements in a test agent (PAPE)‐treated samples are divided by the average of control samples (at least six vehicle controls from the same plate) to generate a ratio that is then log10 transformed. Significance prediction envelopes are calculated using historical vehicle control data at a 95% confidence interval. As a part of the assay acceptance evaluation, the QA/QC Pearson Test is performed by first establishing the 1% false negative Pearson cutoff from the historical positive controls reference dataset. The process iterates through every profile of system biomarker readouts in the positive control reference dataset, calculating Pearson values between each profile and the mean of the remaining profiles in the dataset.

The overall number of Pearson values used to determine the 1% false negative cutoff is the total number of profiles present in the reference dataset. The Pearson value at the one percentile of all values calculated is the 1% false negative Pearson cutoff. A system will pass if the Pearson value between the experimental plate's negative control or drug control profile and the mean of the historical control profiles in the reference dataset exceeds this 1% false negative Pearson cutoff. Overall assays are accepted when each individual system passes the Pearson test, and 95% of all project plates have % a CV <20%.

### 2.3 In vivo study: a clinical, double‐blind randomized trial

#### 2.3.1 Trial design

The study utilized a double‐blind, randomized, controlled design. Female participants were selected from the Zurko Research database in Madrid, Spain, during September and October 2023. Selection criteria included age, skin type, and skin condition. Subsequently, participants were randomly allocated to one of two groups—Group A used Product A and Group B used Product B. Evaluations were conducted at the start of the trial and again after 28 days to assess changes within each subject.

#### 2.3.2 Participants

Inclusion criteria included female gender, age 30–70 years old, all skin types, dry skin condition, with visible age signs. Exclusion criteria comprised the presence of pathologies in the experimental area and any pathologies that might interfere with the study, pregnancy or breastfeeding, immunodeficiency, history of allergies, photosensitivity, or phototoxicity, intense sunlight or UV exposure during the study or taking sunbaths or UV rays during the month before the study in the test area, treatments with antibiotics, antihistamines, corticosteroids, beta‐blockers, retinoid, azelaic acid, anti‐acne treatments or for which treatment is completed during the 15 previous days to the study, application of other similar topical products in the experimental area during the study, or application of other cosmetic types not used usually in the experimental area.

Forty‐seven female participants were included in the study, aged 32–68 years old. The number of participants required at the end of the study was 40; 20 were required for Product A, and 20 were required for Product B. 60% of participants had sensitive, and 40% had non‐sensitive skin. They have been randomized to groups A and B according to the products used (Product A and Product B).

#### 2.3.3 Experimental procedure and outcomes

Products were applied twice daily, using two to four pea‐sized amounts in the morning and evening, to the clean face and neck area.

Measurements were done on Days 0 and 28 in a controlled environment with a temperature range of 18°C −23°C and relative humidity between 40% and 60%. Participants acclimated to these conditions for 20–30 min before measurements were taken to standardize results.

To assess the product's anti‐wrinkle efficacy, the PRIMOS‐CR® device was employed at the beginning of the study (D0) and after 28 days (D28). PRIMOS‐CR® is an optical system that measures wrinkles on the skin's surface using a pattern of parallel bands projected by a digital projector. The data collected by a high‐speed camera is sent to a computer for analysis. Wrinkles are assessed by the parameter Ra, which calculates the average of the single roughness depths. The lower the value Ra, the less wrinkle depth, length, and volume, indicating better anti‐wrinkle efficacy. In this study, wrinkles were measured in the crow's feet area (outer corner of eyes).

A dermatologist conducted a clinical evaluation at the start and after 28 days of continuous product use, focusing on the ocular area and potential alterations or pathologies. This comprehensive assessment included a visual evaluation and detailed documentation of any changes. Using a five‐point scale to grade various skin conditions such as desquamation, dryness, acne, redness, spots, edema, and vesicles, the dermatologist also determined the likelihood of these alterations being related to product use on a six‐point scale.

After 28 days of product use, participants filled out a questionnaire regarding the product's organoleptic characteristics, packaging, perceived efficacy, purchase intention, and willingness to use the product in the future. Organoleptic characteristics and efficacy were rated on a five‐point scale, ranging from strong dislike to strong preference or agreement.

In instances of unwanted symptoms, a specialist completed an Unwanted Effects Notification Form to document and evaluate adverse reactions, ensuring the safety and well‐being of participants throughout the study.

### 2.4 Data analysis

The model used for data analysis is the lme function of the nlme package in R. If data follow a normal distribution, a Student's *t*‐test is used, otherwise, the Wilcoxon signed‐rank test is used. A significance level of 0.05 was established for each test, and they were performed for each participant group (Group A and Group B). If the null hypothesis is rejected, it can be concluded that there are significant differences in treatment.

## 3 RESULTS

### 3.1 PAPE characterization

The extraction process yielded 35.3% of propolis in the initial extract. Figure 1 shows the initial extracts chromatographic profile and concentrations of relevant markers of biological activity, respectively.

**FIGURE 1:**
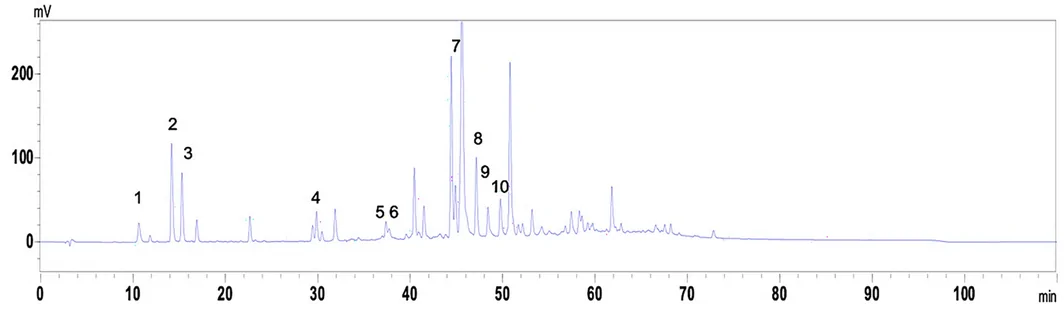
Chromatographic profile of PAPE, with 10 markers of biological activity, obtained by HPLC‐UV method^18^, ^19^: (1) caffeic acid, (2) *p*‐coumaric, (3) ferulic acid, (4) *trans*‐cinnamic, (5) kaempferol, (6) apigenin, (7) chrysin, (8) pinocembrin, (9) CAPE, (10) galangin.

All 10 markers of biological activity were identified and quantified. Ferulic, para‐coumaric, and caffeic acid were the most abundant compounds in the extract.

Table 1 shows the concentrations of markers in both the initial extract and the standardized extract, PAPE. PAPE is used in in vitro studies and clinical trials. The concentrations shown in Table 1 correspond to Product B with 3% extract.

**TABLE 1:** Concentrations of markers of biological activity in the initial extract and PAPE (μg/g).

Marker	Initial extract (μg/g)	PAPE (μg/g)
Caffeic acid	1627.50	218.08
*Para‐*coumaric acid	4471.54	524.74
Ferulic acid	4715.49	544.06
CAPE	3269.00	31.55
*Trans‐*cinnamic acid	550.93	48.55
Kaempferol	735.12	24.72
Apigenin	1494.96	22.40
Chrysin	3566.04	13.92
Pinocembrin	10337.01	82.61
Galangin	4784.02	26.54
The sum of 4 relevant markers	14128.53	1318.43

As shown in Table 1, the sum of 4 markers relevant to the PAPE standardization was 1318.43 μg/g.

### 3.2 In vitro study–BioMAP profiling

The highest sum of the phenolic marker concentration used in in vitro tests was 164.8 μg/g. Results of BioMAP profiling in two cell‐based systems are shown in Figures 2 and 3. The evaluation within the HDF3CGF and KF3CT BioMAP systems revealed distinct changes in critical biomarkers related to skin biology.

**FIGURE 2:**
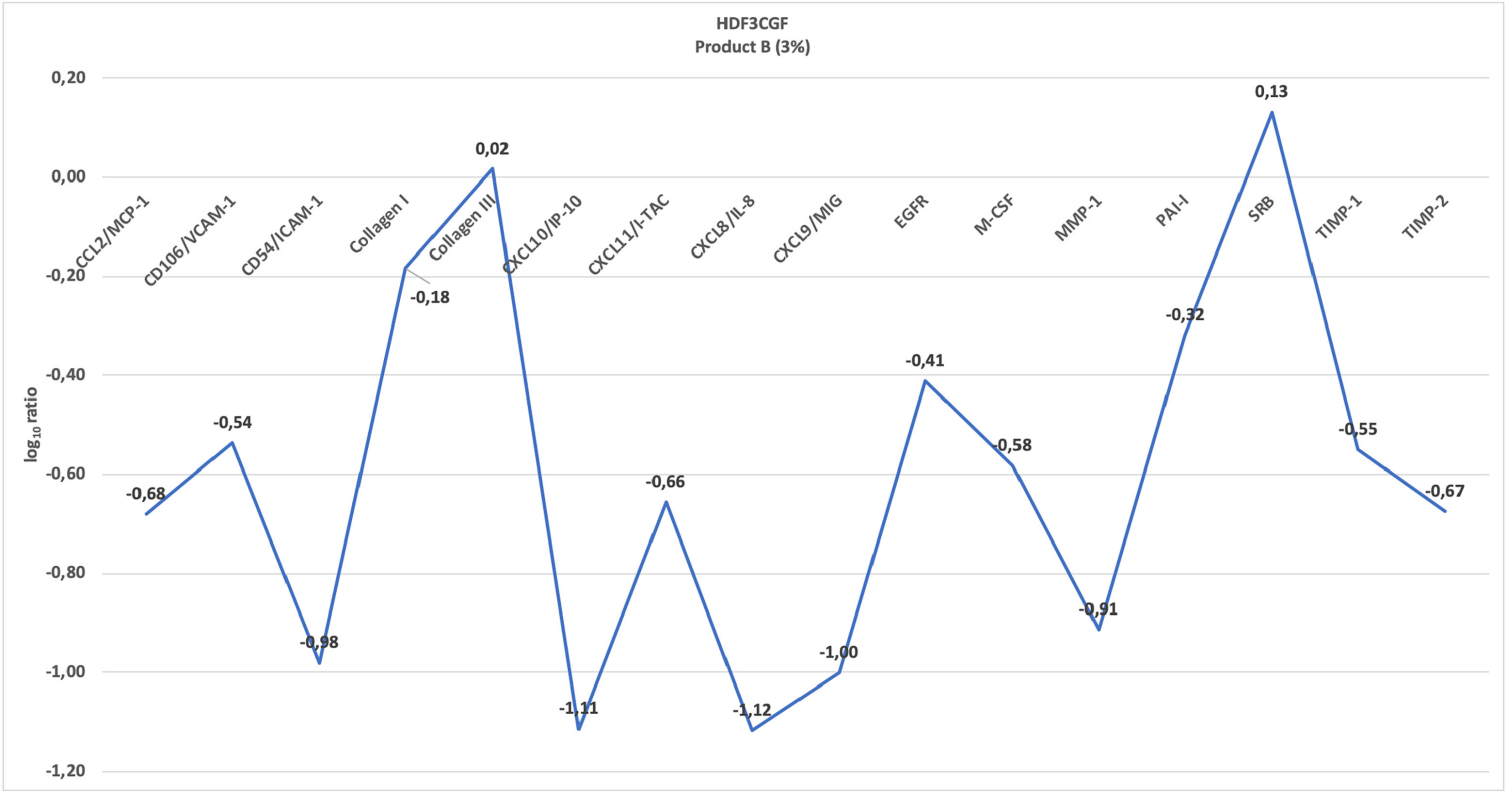
PAPE profile in HDF3CGF system. The x‐axis shows the quantitative protein‐based biomarker readouts. The y‐axis represents a log‐transformed ratio of the biomarker readouts for the drug‐treated sample (*n* = 1) over vehicle controls (*n* ≥ 6). Biomarker activities are annotated when two or more consecutive concentrations change in the same direction relative to vehicle controls, are outside the significance envelope, and have at least one concentration with an effect size >20% (|log10 ratio|>0.1).

**FIGURE 3:**
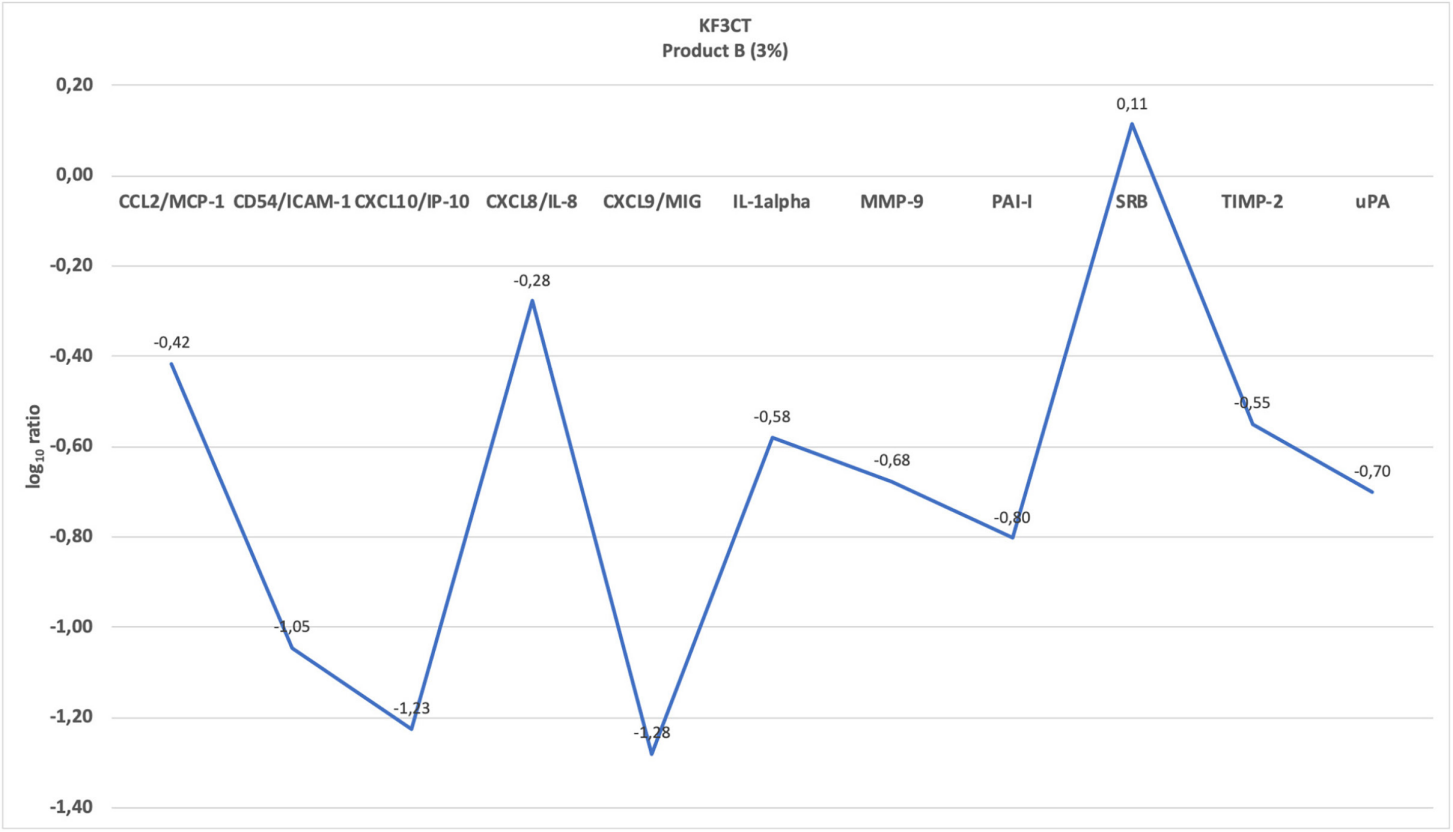
PAPE BioMAP profile in KF3CT system. The x‐axis shows the quantitative protein‐based biomarker readouts. The y‐axis represents a log‐transformed ratio of the biomarker readouts for the drug‐treated sample (*n* = 1) over vehicle controls (*n* ≥ 6). Biomarker activities are annotated when two or more consecutive concentrations change in the same direction relative to vehicle controls, are outside the significance envelope, and have at least one concentration with an effect size >20% (|log10 ratio|>0.1).

In HDF3CGF system, the decreased activity was observed in biomarkers: MCP‐1 (CCL2/MCP‐1), VCAM‐1 (CD106/VCAM‐1), ICAM‐1 (CD54/ICAM‐1), IP‐10 (CXCL10/IP‐10), I‐TAC (CXCL11/I‐TAC), IL‐8 (CXCL8/IL‐8), MIG (CXCL9/MIG), M‐CSF, MMP‐1, and PAI‐I. An increase was noted in Collagen III and the proliferation marker SRB. TIMP‐1 and TIMP‐2 both showed decreased activity.

In KF3CT System the decreased activity was seen in MCP‐1 (CCL2/MCP‐1), ICAM‐1 (CD54/ICAM‐1), IP‐10 (CXCL10/IP‐10), IL‐8 (CXCL8/IL‐8), MIG (CXCL9/MIG), IL‐1α, MMP‐9, PAI‐I, and TIMP‐2. There was an increase in SRB and a decrease in uPA.

### 3.3 In vivo study: a clinical, double‐blind randomized trial

#### 3.3.1 Anti‐wrinkle efficacy

Table 2 shows the products' anti‐wrinkle efficacy. Twenty‐eight days after the continuous use of Product A (1.5% PAPE), anti‐wrinkle efficacy improves by an average of 25% in relation to the baseline (*p* = 0.004). Twenty‐eight days after the continuous use of Product B (3% PAPE), anti‐wrinkle efficacy improves by an average of 34% in relation to the baseline (*p* = 0.004).

**TABLE 2:** Descriptive analysis and significance for the anti‐wrinkle efficacy (*n* = 40).

Anti‐wrinkle efficacy	D0	D28
Product A—PAPE 1.5%		
Average Ra value (μm)	5.76	4.31
Standard deviation	3.80	2.13
% of absolute variation respect to D0		−25%
% of panelists with improvement		70%
Wilcoxon signed‐rank test (D28 vs. D0)		
V		182.00
*p* value		0.004
Significance		S
Product B—PAPE 3%		
Average Ra value (μm)	7.70	5.09
Standard deviation	5.31	2.50
% of absolute variation respect to D0		−34%
% of panelists with improvement		75%
Wilcoxon signed‐rank test (D28 vs D0)		
V		185.00
*p* value		0.003
Significance		S

The images of the participant's crow's feet area on Days 0 and 28 taken with PRIMOS‐CR® are shown in Figures 4 and 5.

**FIGURE 4:**
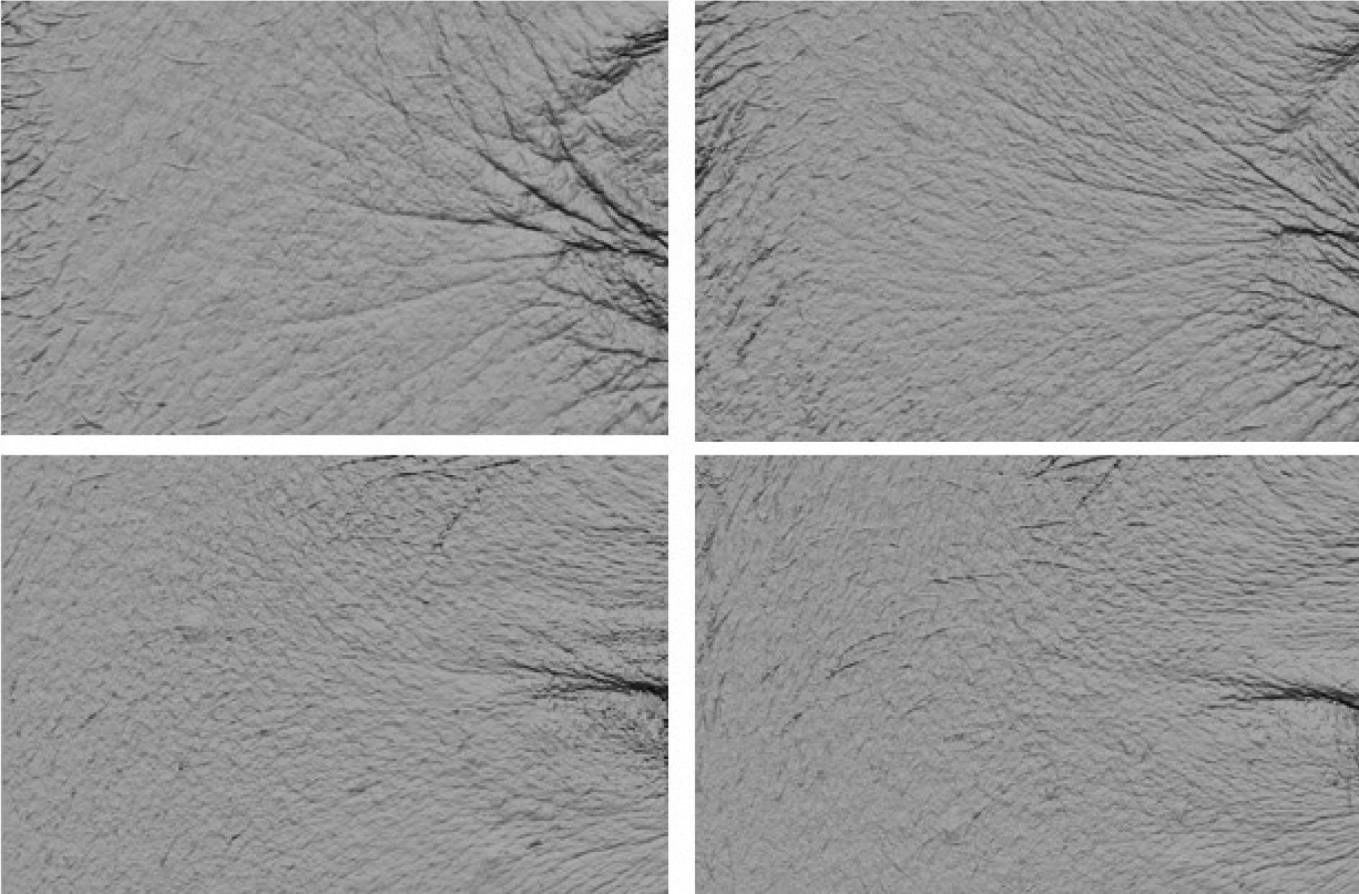
The images of the two participant's crow's feet area on Days 0 (left) and 28 (right) taken with PRIMOS‐CR®.

**FIGURE 5:**
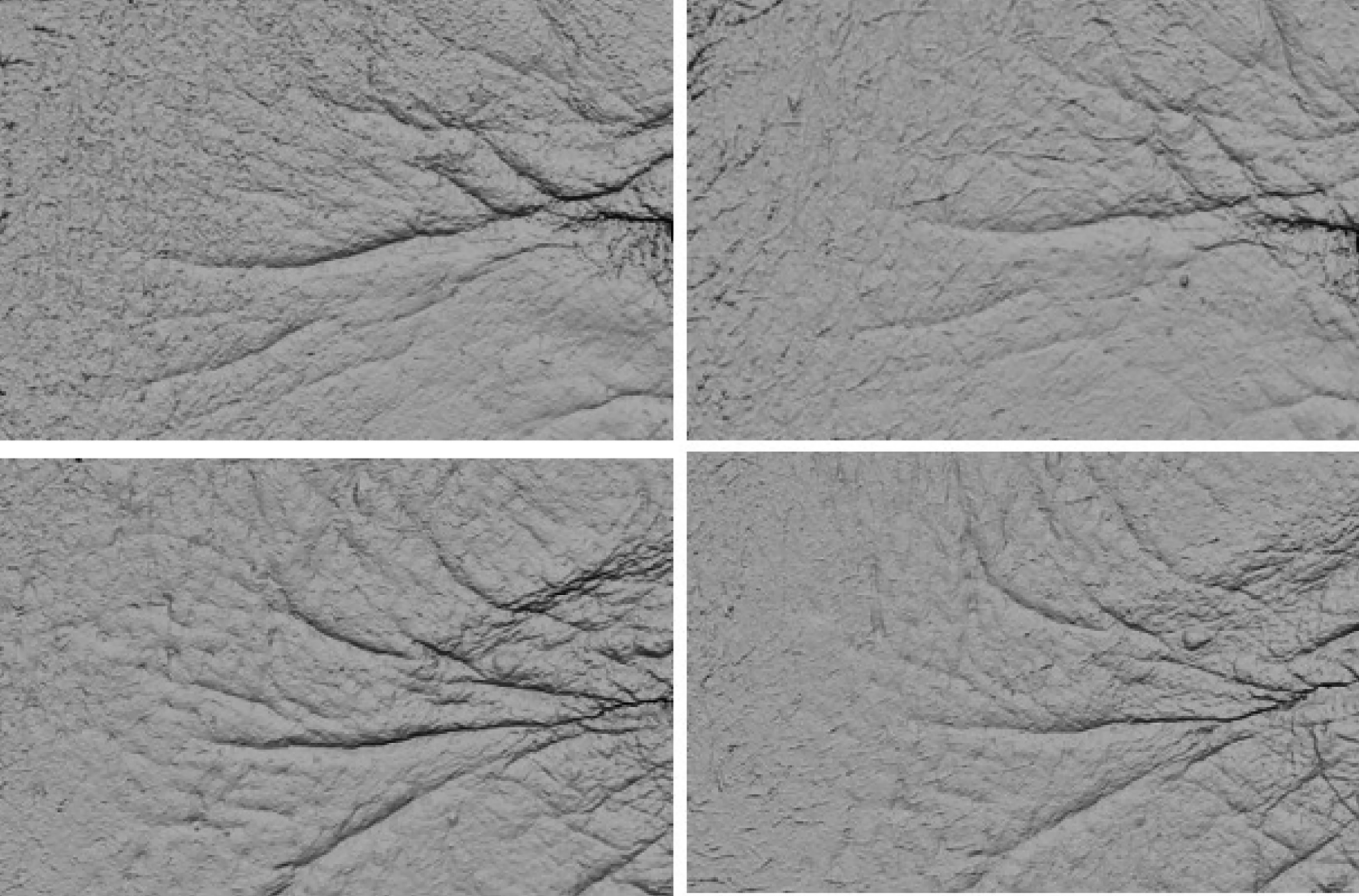
The images of the two participant's crow's feet area on Days 0 (left) and 28 (right) taken with PRIMOS‐CR®.

#### 3.3.2 Subjective effectiveness

According to the results presented in Table 3, after 28 days of continuous use of Product A, 90% of the participants were satisfied with the product overall, and the participants said that they would continue applying this product.

**TABLE 3:** Subjective effectiveness of Product A and Product B (% of participants).

Subjective effectivness	Product A: PAPE 1.5%	Product B: PAPE 3%
After the use of the product, I feel an antiaging effect	85%	75%
Would continue applying this product	95%	85%
After the use of the product, I feel my skin more luminous and radiant	90%	90%
After the use of the product, I feel my skin Is protected against external agents	75%	85%
After the use of the product, I feel like the product smooths and reduce the the wrinkles and expression lines	75%	75%
Extensibility on the skin	100%	85%
After the use of the product, I feel the skin firmer	85%	85%
After the use of the product, I feel the skin moisturized	90%	95%
Purchase intention (surely yes and probably yes)	85%	85%
Expectations met	80%	85%

After 28 days of continuous use of Product B, 80% of the participants were satisfied with the product, and 85% of participants said that they would continue applying this product.

#### 3.3.3 Adverse reaction after the use of the product

According to the clinical examination performed by the dermatologist, none of the participants showed any skin alteration at 56 days associated with the use of Product A and Product B.

## 4 DISCUSSION

Exploring the role of phenolic acids in anti‐wrinkle applications offers a promising avenue for skincare research. Known for their antioxidant, anti‐inflammatory, and MMP‐inhibitory properties, phenolic acids also penetrate the skin effectively and provide photoprotective benefits, positioning them as ideal components in advanced antiaging skincare formulations.^7^

Chromatographic analysis has revealed that propolis extract is a rich source of a broad spectrum of phenolic compounds, including notable acids such as caffeic, ferulic, p‐coumaric acid, and CAPE. Significantly, the extract boasted 4715.49 μg/g of ferulic acid, surpassing the 3000 μg/g found in whole bran,^28^ and contained 1627.50 μg/g of caffeic acid, which is notably higher than the 175–875 μg/g found in coffee.^29^, ^30^ This broad spectrum and high content of phenolic compounds highlight propolis extract's substantial potential for varied activities in skin care.

In vitro studies on phenolic acid polymer extract (PAPE) demonstrated its regulatory effects on several inflammation and tissue remodeling biomarkers. For instance, in the HDF3CGF system, reductions in monocyte chemoattractant protein 1 (MCP‐1), vascular cell adhesion molecule 1 (VCAM‐1), intracellular cell adhesion molecule 1 (ICAM‐1), and matrix metalloproteinase 1 (MMP‐1) suggest a significant anti‐inflammatory impact and a possible improvement in skin structure and function, as evidenced by the increase in collagen III levels and SRB activity, which indicates enhanced matrix synthesis or stabilization and potential benefits for cellular growth and viability. The decreased activity of tissue inhibitors of metalloproteinase (TIMP) 1 and 2 further supports the idea of PAPE influencing tissue repair processes.

Similarly, findings from the KF3CT system showed that PAPE could decrease inflammatory markers like MCP‐1, ICAM‐1, and interleukin 8 (IL‐8), along with MMP‐9, indicating a pronounced anti‐inflammatory and anti‐degradative effect on the extracellular matrix. The reduction in PAI‐I activity points to a modulation of proteolytic activity, aiding in maintaining extracellular matrix integrity. An increase in SRB activity suggests a role in promoting keratinocyte proliferation, essential for skin regeneration. These results align with studies confirming the anti‐inflammatory properties of various propolis extracts and phenolic acids.^11^

The in vitro findings correspond with the clinical observations, where PAPE achieved a statistically significant dose‐dependent reduction in wrinkles in the crow's feet area, demonstrating dose‐dependent effects of 25% and 34%.

The effectiveness of PAPE can be compared to other known anti‐wrinkle compounds, such as topical retinoids and bakuchiol. Tretinoin is the topical retinoid with the strongest clinical evidence and a gold standard of antiaging activity.^31^ After 8 weeks of a 0.02% tretinoin regimen, there was an average of 11% reduction in wrinkle area.^32^ In a study with double conjugated retinoids, wrinkles were significantly reduced by 14.3% at 4 weeks, 23.8% at 8 weeks, and 33.3% at 12 weeks.^33^ A comparative study of the effects of retinol and retinoic acid reported a reduction of 27.93% in wrinkles in the eye area after 4 weeks of 0.1% retinol treatment and 38.74% after 12 weeks of treatment.^1^ In a study conducted to determine the effectiveness and effect rate of retinol concentrations ranging from 1500 to 6600 IU, it was found that after 24 weeks of treatment with different concentrations, there was a 30% improvement on average in the area of crow's feet among those using the 6600 IU dosage and a 21% improvement among those using the 1500 IU dosage of retinol.^34^ Retinoids are known to reduce signs of aging effectively but can cause skin irritation,^35^ ocular discomfort, and dry eyes when used around the eyes.^36^ For this reason, researchers are extensively studying safer alternatives. Bakuchiol is a natural compound that has been shown to have antiaging effects comparable to retinol. In fact, a study reported a statistically significant decrease of 11% in wrinkles after 12 weeks of bakuchiol application. This compound functions as an antiaging agent through gene expression regulation similar to retinol.^37^

Our results show that PAPE is comparable in effectiveness to topical retinoids and bakuchiol: wrinkles were reduced by 34% after using face cream with 3% PAPE for only 4 weeks and 25% when using 1.5% PAPE.

The exploration into natural bioactive compounds for combating the signs of skin aging, particularly through the lens of chronic inflammation, marks a pivotal advancement in dermatological research and cosmetic innovation. PAPE is a complex mixture of reproducible phenolic compound content,^18^ but recent proteomic analysis has demonstrated it contains proteins as well, adding another layer to its efficacy.^38^ This discovery suggests that protein or peptide activity could further contribute to its antiaging effects, opening new avenues for further research.

Most importantly, shows a favorable safety profile with no reported negative side effects, making it suitable for both sensitive and nonsensitive skin types as a tolerable and gentle antiaging solution. This approach not only illuminates a path for visibly reducing skin roughness and wrinkles but also aligns with a deeper understanding of skin aging as a process intricately tied to chronic inflammatory mechanisms and meets the growing consumer demand for effective and gentle products.

## CONFLICT OF INTEREST STATEMENT

Bozo Radic and Jelena Suran are shareholders and, along with Sasa Radic, are employees of Apitox Technologies, which owns the patent No. WO/2020/169425, Liquid propolis extract, its formulation and the use thereof.

## 5 ETHICS STATEMENT

The research adhered to the ethical standards stipulated in the most recent iteration of the Declaration of Helsinki. Prior to initiation, all participants were thoroughly informed about the nature and procedures of the study, subsequently providing their written informed consent. These consent documents are securely archived at Zurko Research (Zurko Research S.L., Madrid, Spain).

## Data Availability

The data that support the findings of this study are available from the corresponding author upon reasonable request.
